# *Cutis verticis gyrata*: a cutaneous finding in acromegaly^☆^

**DOI:** 10.1016/j.abd.2021.05.017

**Published:** 2022-03-11

**Authors:** Giullia Menuci Chianca Landenberger, Bárbara Roberta Ongaratti, Júlia Fernanda Semmelmann Pereira-Lima, Miriam da Costa Oliveira

**Affiliations:** a Neuroendocrinology Center, Complexo Hospitalar Santa Casa de Porto Alegre, Universidade Federal de Ciências da Saúde de Porto Alegre, Porto Alegre, RS, Brazil; b Postgraduate Program in Pathology, Universidade Federal de Ciências da Saúde de Porto Alegre, Porto Alegre, RS, Brazil

**Keywords:** Acromegaly, Growth hormone, Scalp

## Abstract

Acromegaly is a rare disease characterized by changes in the bone and soft tissue systems, induced by excess growth hormone and insulin-like growth factor type 1. Among the skin lesions associated with acromegaly is cutis verticis gyrata, an hypertrophic, and coarse folding of the skin of the scalp, an association of uncommon incidence and unknown prevalence. This case report describes the case of a patient diagnosed with acromegaly at age 60 with previously unidentified cutis verticis gyrata. This report aims to review the literature on cutis verticis gyrata and its unusual association with acromegaly.

## Case report

A 60-year-old black male was referred to a tertiary outpatient neuroendocrinology center for suspected acromegaly. He had type 2 diabetes mellitus, diagnosed at age 40, with diabetic neuropathy and retinopathy, and was treated with insulin and metformin. He had systemic arterial hypertension and dyslipidemia. His shoe size had increased by age 40. He reported a femoral neck fracture at age 53 after a fall, and bone mineral densitometry showed osteoporosis of the lumbar spine. The family history included diabetes mellitus and obesity.

**Figure 1:**
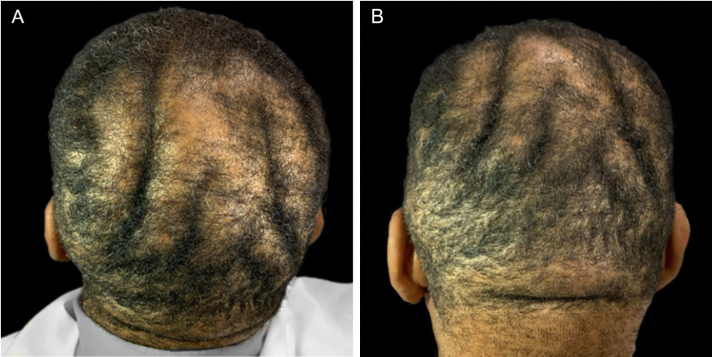
(A and B) Clinical aspect of the scalp.

**Figure 2:**
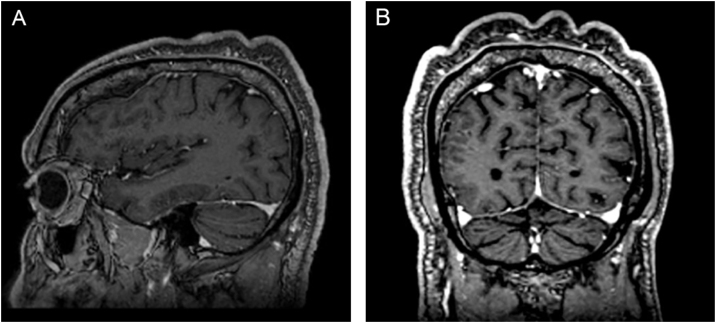
Magnetic resonance imaging of the skull. (A), Sagittal section. (B), Coronal section, showing skin undulations.

**Figure 3:**
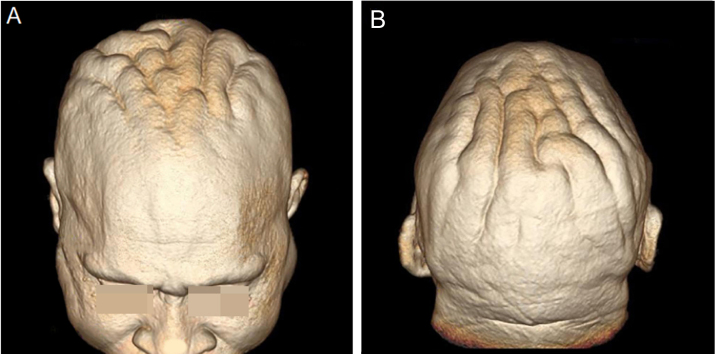
(A and B), Three-dimensional (3D) reconstruction of the cerebriform skin from the magnetic resonance imaging of the cephalic segment.

On physical examination, his height was 182 cm, BMI of 26.1 kg/m²; he had prognathism and enlargement of the extremities, increased skin folds and furrows, increased skin sweating, percussion of the median nerves resulted in bilaterally positive Tinel sign, and he had a deep voice. Convolutions of the scalp were observed in the parietal and occipital regions (Fig. 1). The alterations observed on the scalp were also identified in the magnetic resonance imaging of the skull (Figure 2, Figure 3). When questioned, the patient reported that his longtime barber reported having difficulty cutting his hair due to "irregularities" on the scalp.

The diagnosis of acromegaly was confirmed by the insulin-like growth factor 1 (IGF-1) level of 734 ng/mL (reference interval 81‒225 ng/mL) and growth hormone (GH) of 21.5 ng/mL (reference range <3 ng/mL). There was no clinical or laboratory evidence of hypopituitarism. Magnetic resonance imaging of the sellar region showed a pituitary lesion to the right, measuring 1.3 × 0.7 cm in the longest axis and involving the right cavernous sinus. The patient underwent transsphenoidal pituitary resection, with pathological confirmation of a pituitary adenoma, reactive on immunohistochemistry for GH, prolactin, and thyroid stimulating hormone (TSH), and positive Ki-67 immunoreactivity in 2% of the neoplastic cells. Due to the residual lesion and persistence of GH/IGF-1 hypersecretion, he started using 30 mg of somatostatin analog (octreotide) per month after biochemical control of the excess hormone.

## Discussion

Cutis verticis gyrata (CVG) is the term that describes hypertrophy and coarse folding of the scalp skin, alternating crests and deep grooves, which mimic a cerebriform aspect, that is, the cerebral gyri. CVG is classified as primary when the etiology is unknown or has a neurological cause. Acromegaly is one of the secondary causes of CVG, as well as local scalp diseases, pachydermoperiostosis, genetic syndromes, systemic diseases, other endocrine diseases, and minoxidil or testosterone use.^1^, ^2^, ^3^ CVG is a rare condition, with a prevalence of 0.026 to 0.1 in 100,000 individuals, and reports of its association with acromegaly are even more rare.^4^

The number of folds, usually soft and spongy, can vary.^1^ Skinfolds in primary CVG are usually symmetrical, follow an anteroposterior direction, and usually involve the vertex and occiput, although the entire scalp can be affected. In secondary CVG, the folds are often asymmetrical, not following a longitudinal direction.^5^ The folds are not easily flattened by traction or pressure.^3^, ^6^ The areas of the scalp affected in acromegaly are multiple, ranging from frontal, frontoparietal, and parieto-occipital regions, up to "forehead to nape of the neck" and, eventually, they affect the glabella and nasal bridge.^1^, ^2^, ^3^, ^5^, ^6^, ^7^, ^8^

While males are vastly more affected in cases of primary CVG, at a ratio of 5-6:1, this predominance is not well established in secondary cases.^9^

Schunter et al. proposed that excessive levels of GH and IGF-1 are crucially involved in the pathogenesis of CVG in acromegaly, acting on target skin cells, especially dermal fibroblasts.^7^

The collagen deposition that characterizes CVG is prolonged and progressive, but it is a benign condition, with repercussions limited to esthetics – consequently, with possible emotional damage and eventual skin infections, mainly fungal, due to the difficult hygiene of the furrows. The treatment consists of local hygiene and surgical resection of excess skin in cases of cosmetic discomfort.

The current case does not require specific management for CVG, but draws attention to the possibility of this complication in the context of a rare and chronic disease such as acromegaly.

## Financial support

None declared.

## Authors' contributions

Giullia Menuci Chianca Landenberger: Approval of the final version of the manuscript; design and planning of the study; drafting and editing of the manuscript; collection, analysis, and interpretation of data.

Bárbara Roberta Ongaratti: Approval of the final version of the manuscript; critical review of the literature; critical review of the manuscript.

Júlia Fernanda Semmelmann Pereira-Lima: Approval of the final version of the manuscript; effective participation in research orientation; intellectual participation in the propaedeutic and/or therapeutic conduct of the studied cases.

Miriam da Costa Oliveira: Approval of the final version of the manuscript; design and planning of the study; critical review of the manuscript.

## Conflicts of interest

None declared.
